# Down-selection of the VAR2CSA DBL1-2 expressed in *E. coli* as a lead antigen for placental malaria vaccine development

**DOI:** 10.1038/s41541-018-0064-6

**Published:** 2018-07-17

**Authors:** Arnaud Chêne, Stéphane Gangnard, Célia Dechavanne, Sebastien Dechavanne, Anand Srivastava, Marilou Tétard, Sophia Hundt, Odile Leroy, Nicolas Havelange, Nicola K. Viebig, Benoît Gamain

**Affiliations:** 1 Université Sorbonne Paris Cité, Université Paris Diderot, Inserm, INTS, Unité Biologie Intégrée du Globule Rouge UMR_S1134, Severe Malaria Pathogenesis group, Laboratoire d’Excellence GR-Ex, Paris, France; 2 European Vaccine Initiative, UniversitätsKlinikum Heidelberg, Voßstraße 2, 69115 Heidelberg, Germany

## Abstract

Over 50 million women are exposed to the risk of malaria during pregnancy every year. Malaria during pregnancy is a leading global cause of maternal morbidity and adverse pregnancy outcomes. Adhesion of *Plasmodium falciparum*-infected erythrocytes to placental chondroitin-4-sulfate (CSA) has been linked to the severe disease outcome of placental malaria. Accumulated evidence strongly supports VAR2CSA as the leading placental malaria vaccine candidate. Recombinant proteins encompassing the VAR2CSA high affinity CSA binding site have been generated, and their activity as immunogens that elicit functional (inhibitory) and cross-reactive antibodies against CSA-binding parasites assessed. The expression of His-tagged proteins was compared in four different expression systems and their capacity to bind specifically to CSA was analyzed. CHO cells and *E. coli* SHuffle cells were the two expression systems able to express some of the recombinant proteins in reasonable amounts. Larger analytical scale production of DBL1x-2× (3D7) and DBL3x-4ε (FCR3) best expressed in CHO and *E. coli* SHuffle cells were performed. Purified proteins were administered to rats either alone or adjuvanted with human approved adjuvants. Analysis of the functionality and cross-reactivity of the induced antibodies allowed us to down-select the DBL1x-2(3D7) expressed in *E. coli* SHuffle cells as the best antigen to be transitioned to further clinical development in order to protect future pregnant women living in malaria endemic areas against the severe clinical outcomes of placental malaria.

## Pre-clinical studies: Finding the tools to beat placental malaria

A mix of the right parasitic protein with the right production method has yielded a vaccine candidate for placental malaria. Primarily affecting first-time pregnant women, placental malaria is estimated to cause 200,000 infant deaths and 10,000 maternal deaths annually. In this study, led by Benoît Gamain, researchers from France’s INSERM and Germany’s European Vaccine Initiative assayed a combination of proteins designed to target and block a key pathogenic mechanism of parasite-infected red blood cells. Finding the highest performing protein, the researchers also used an *Escherichia coli* expression system able to replicate and fold the complex protein correctly. During tests, this protein/vector combination bested others in production qualities and immunogenicity. The team’s efforts laid the foundations for a scalable, low-cost vaccine that is currently undergoing clinical trials.

## Introduction

Malaria caused by *Plasmodium* constitutes a major health problem and is still one of the most common deadly infectious diseases in the world. In 2016, the global tally of malaria reached 216 million cases and 445,000 deaths, a large majority of them resulting from *Plasmodium falciparum* infection.^1^ Individuals living in high *P. falciparum* transmission settings gradually acquire immunity to the most severe clinical manifestations of the infection. However, regardless of previous exposure to the parasite, first time pregnant women (primigravidae) are susceptible to placental malaria (PM) with severe adverse consequences such as: maternal anemia, fetal growth retardation and preterm delivery.^2^ Each year, PM is responsible for 20% of stillbirths in sub-Saharan Africa, 11% of all newborn deaths in sub-Saharan Africa, and 10,000 maternal deaths globally.^3–5^

Remarkably, multigravid women are at lower risk of developing PM than primigravid women, highlighting that clinical immunity against PM develops progressively.^2^ This gradual protection has been associated with the acquisition of antibodies that recognize the surface of infected erythrocytes from placental origin and block their adherence to the syncytiotrophoblastic lining of the placenta.^6–8^ This natural acquisition of immunity is nourishing hope for the development of a vaccine that would protect pregnant women the fetus and the newborn against the dire outcomes of PM.

To escape immune responses and spleen dependent clearance, *P. falciparum* has developed efficient immune evasion strategies such as antigenic variation and cytoadhesion mechanisms which play a central role in pathogenicity.^9^ Cytoadhesion of *P. falciparum*-infected erythrocytes (IEs) is mediated by members of the highly diverse *P. falciparum* erythrocyte membrane protein 1 (PfEMP1) family encoded by approximately 60 *var* genes per parasite genome.^10^

IEs isolated from placenta present a distinct adhesive phenotype, as they do not bind to the common receptors used by the parasite to adhere to the microvasculature walls such as CD36 or ICAM-1.^6,11^ Instead, they are able to bind to the sulfated glycosaminoglycan chondroitin sulfate A (CSA).^12^ CSA-proteoglycans are found on a large variety of cell types in the body but can functionally differ depending on the core protein on which the CSA is attached to, but also on the length and the degree of sulfation of the polysaccharide chain.^13^ Chondroitin proteoglycans appear in the placental intervillous space by the end of the third month of gestation,^14^ thus offering a potential attachment point for IEs. The multi-domain PfEMP1 protein, VAR2CSA, has been identified as the major parasite-derived ligand mediating IEs adhesion to the placental receptor CSA.^15–17^

The VAR2CSA protein is a 350 kDa transmembrane protein, with a 300 kDa extracellular region, comprising six DBL (Duffy-binding-Like) domains and four interdomain regions (INT). Its native conformation seems to require a complex folding process and the formation of many disulfide bonds between paired-cysteines.^18^ Its large size and structural complexity make the full-length protein difficult to express and likely incompatible with large-scale production for vaccination strategies. Therefore, as the mechanism of PM protection is thought to be mediated primarily by blocking IEs adhesion to the syncytiotrophoblastic lining of the placenta, research on VAR2CSA-based vaccine candidates was first oriented to the identification of the minimal CSA-binding region of the protein that would retain complete functionality and display conserved native antigenic sequences in order to induce cross-reactive and cross-inhibitory antibodies. The full-length extracellular part of VAR2CSA expressed as a recombinant protein was shown to bind to CSA with higher specificity and affinity than individual domains,^18,19^ highlighting the possible presence of a binding pocket comprising multi-domains in the native protein. This was later confirmed by the identification of the N-terminal region of the protein (DBL1x-3×) as the minimal CSA-binding region,^20^ the binding site being more precisely located within the ID1-ID2a sequence.^19^ These results reshaped our view on PM vaccine design and let us envisaged the use of multi-domain constructs of limited size that would generate antibodies able to block the adhesion of IEs to the placenta, notably by (i) targeting residues directly involved in CSA-binding and/or (ii) targeting epitopes in close proximity of the CSA-binding sites and thus possibly restraining CSA accessibility by steric hindrance.

The feasibility demonstration of such vaccines could arise from ongoing VAR2CSA-based developmental vaccine initiatives such as PAMVAC and PRIMALVAC.^21^ The main objective of our work within the PRIMALVAC project is therefore to obtain a proof of concept that a VAR2CSA-based vaccine can be designed for human use and can induce long lasting or rapidly boosted immune responses producing strain-transcending and cross-inhibitory antibodies crucial for clinical protection.

In order to bring a VAR2CSA-based vaccine candidate to phase Ia/Ib clinical trial, it is critical to identify in early phase of development, a suitable heterologous expression system, thus allowing (i) the production of sufficient amount of recombinant proteins to permit pre-clinical testing and (ii) a relatively straightforward transition to current good manufacturing practice (GMP). Many studies described the recombinant protein production of different truncated forms of the VAR2CSA extracellular part in various heterologous expression systems, not always fully functional and most often with highly variable yields not suitable for large-scale vaccine production.^20,22–24^

This study describes the down-selection of a VAR2CSA-based vaccine candidate, which would be able to generate antibodies capable of (i) cross-reacting with native VAR2CSA expressed at the surface of erythrocytes infected with different parasite strains from worldwide origins and (ii) inhibiting the interaction between IEs and CSA. A multi-system expression study was first undertaken to determine the best system-protein combination(s) in terms of productivity and quality of recombinant proteins before pre-clinical immune assessments in rats.

## Results

### Multi-system expression of recombinant proteins

**Fig. 1:**
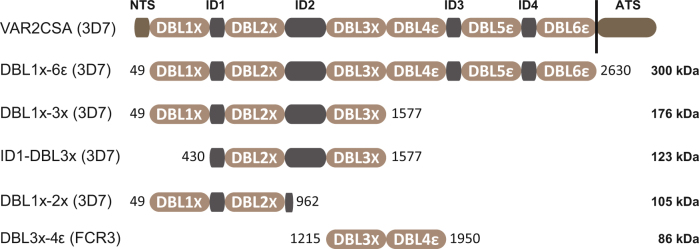
Schematic representation of the VAR2CSA domain organization and sequence boundaries of the multi-domain constructs used in this study; DBL1x-6ε (3D7), DBL1x-3× (3D7), ID1-DBL3x (3D7), DBL1x-2× (3D7), and DBL3x-4ε (FCR3). VAR2CSA comprises an N-terminal sequence (NTS), six Duffy binding-like domains (DBL1x to DBL6ε) interspaced by four inter-domain regions (ID1 to ID4), a transmembrane domain and an acidic C-terminal sequence (ATS). The first and last amino-acids of each construct are depicted as they appear within the exon 2 sequence of VAR2CSA from the 3D7 and FCR3 parasite strains

DNA sequences encoding various VAR2CSA-derived constructs (Fig. 1) were cloned into relevant plasmids for soluble protein expression in four different heterologous systems compatible for potential transfer to GMP grade productions (CHO cells, *Pichia pastoris, Lactococcus lactis* and 3 strains of *Escherichia coli*). Since *P. falciparum* does not possess the capacity to add N-glycosylation moieties to newly synthesized proteins during post-translational modification, potential glycosylation sites were mutated within the sequences of constructs meant to be expressed in eukaryotic systems (CHO and *Pichia pastoris)*. Due to the high molecular weight of the DBL1x-6ε (3D7), expression tests were carried out only in CHO cells. Expression of DBL1x-2× (3D7), DBL1x-3× (3D7), ID1-DBL3x (3D7) (PFL0030c), and DBL3x-4ε (FCR3) (IT4var04) were assessed in all four different heterologous systems.

**Fig. 2:**
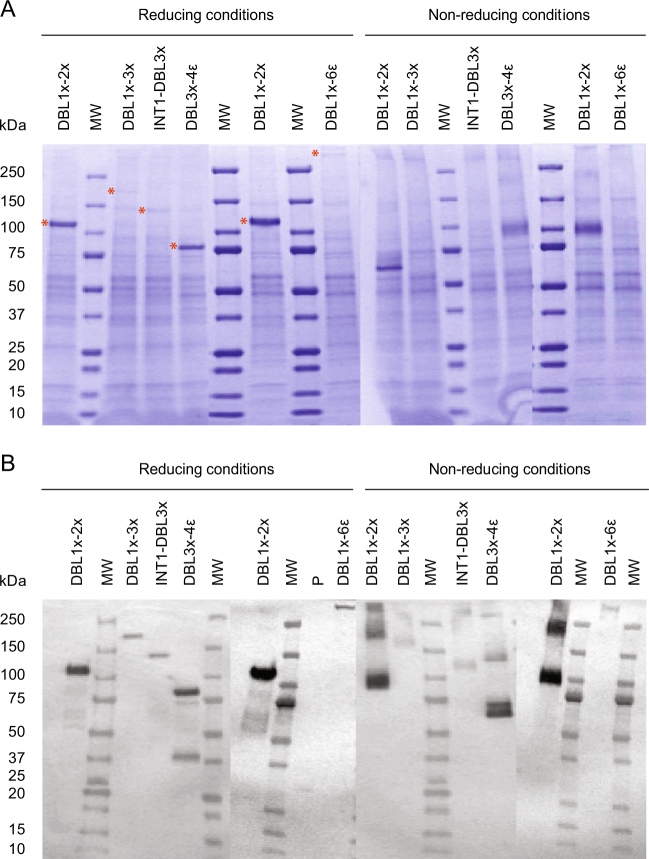
Expression analysis in CHO-Xpress® system of DBL1x-2× (3D7); 105 kDa, DBL1x-3× (3D7); 176 kDa, INT1-DBL3x (3D7); 123 kDa, DBL3x-4ε (FCR3); 86 kDa and DBL1x-6ε (3D7); 300 kDa. **a** Coomassie staining and **b** anti-His western blotting performed on crude culture supernatants after gel electrophoresis in reducing and non-reducing conditions. MW, molecular weight ladder. P, His-tagged protein used as a western blot positive control. All blots derive from the same experiment and were processed in parallel

The full length extracellular region of VAR2CSA, DBL1x-6ε (3D7) as well as the multi-domains DBL1x-2× (3D7), DBL1x-3× (3D7), ID1-DBL3x (3D7), and DBL3x-4ε (FCR3) expressed in the CHO-Xpress® system were all detected in the supernatant of cell cultures. Following gel electrophoresis in denaturing environment (SDS-PAGE) in both, reducing or non-reducing conditions, a band at the expected molecular weight was clearly visible after Coomassie staining for the DBL1x-2× (3D7) and DBL3x-4ε (FCR3) CHO-expressed proteins (Fig. 2a). Subsequent western-blot analysis performed in reducing and non-reducing conditions using an anti-His antibody confirmed the soluble expression of all 5 proteins (Fig. 2b). As the His-tag is located at the C-terminal extremity of the proteins, we observed a minimal amount of N-terminal-truncated protein fragments in reducing conditions. Comparison of protein migration profiles in reducing and non-reducing environments confirmed the formation of disulfide bonds within the recombinant proteins as well as the formation of multimers for DBL1x-2× (3D7) and DBL3x-4ε (FCR3). Estimation of the crude production and purity by capillary electrophoresis revealed relatively modest expression yields (≈5 mg/L) and low purity for DBL1x-6ε (3D7), DBL1x-3× (3D7) and ID1-DBL3x (3D7) in the crude supernatant (Table S1). Nevertheless, DBL1x-2× (3D7) and DBL3x-4ε (FCR3) were expressed at relatively high levels (>65 mg/L) with a decent purity, suitable for subsequent purification steps (Table S1).

**Fig. 3:**
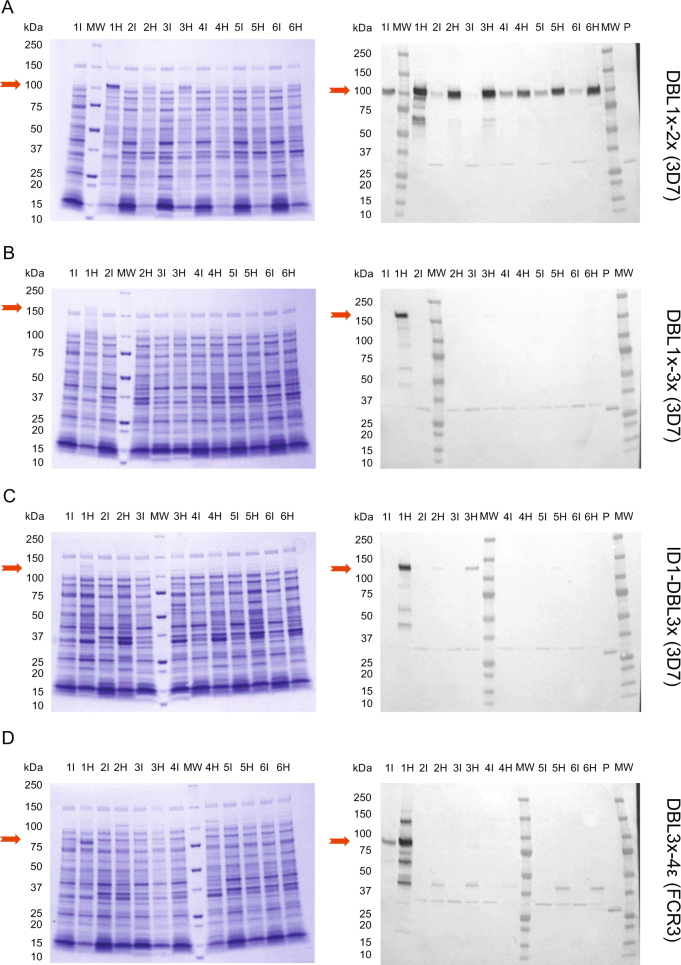
Expression analysis in *E. coli* systems in different test conditions SHuffle express (1), BL21 (2, 4, 5, 6) and HMS174 (3) cells] of **a** DBL1x-2× (3D7), **b** DBL1x-3× (3D7), **c** ID1-DBL3x (3D7), and **d** DBL3x-4ε (FCR3). The left panels represent the Coomassie staining of crude culture supernatants after gel electrophoresis in reducing conditions at the time of induction (I) and at the time of harvesting (H). The right panels display the western blot analysis of protein production using an anti-His antibody. Detailed growth and induction parameters of each expression conditions are depicted in Table S2. Red arrows indicate the expected molecular weights of the proteins of interest. MW, molecular weight ladder. P: His-tagged protein used as a western blot positive control. All blots derive from the same experiment and were processed in parallel

Expression tests in three different *E. coli* strains (BL21, HMS174, SHuffle) were performed for DBL1x-2×(3D7), DBL1x-3×(3D7), ID1-DBL3x (3D7) and DBL3x-4ε (FCR3) in various growth conditions and with diverse induction parameters (Table S2). As described in Table S2, the test conditions 1–3 aimed at comparing strains, the condition 4, at testing an auto-induction and the conditions 5–6, at testing parameters more consistent with industrial scale-up (shorter time and with a small temperature shift). After induction, DBL1x-2× (3D7) was detected in all the soluble fractions obtained after bacteria lysis for all the *E. coli* strains tested (Fig. 3a). DBL1x-3× (3D7), ID1-DBL3x (3D7) and DBL3x-4ε (FCR3) were almost exclusively present in the soluble fractions obtained from the SHuffle system (Fig. 3b–d). The most highly produced proteins were DBL1x-2× (3D7) and then DBL3x-4ε (FCR3) whereas the level of soluble expression of DBL1x-3× (3D7) and ID1-DBL3x (3D7) were lower (Table S1). For all constructs, SHuffle bacteria appeared to be the most appropriate expression system as reflected by a decent crude productivity (>25 mg/L) and purity of these proteins in the cell lysate supernatant (Table S1).

Expression in *Pichia pastoris* was assessed after generating stable recombinant His + Mut + clones. The screening of the best producer clones was done among 250 recombinant clones by Colony lift Immunoassay, revealed with an anti-Flag M2 Dot-Blot. For the DBL1x-3× (3D7) and ID1-DBL3x (3D7) in spite of multiple series of transformation and clone screening, no clones were identified on Dot-Blot, synonymous of no production or low yield. For DBL1x-2× (3D7) and DBL3x-4ε (FCR3), three recombinant protein producing clones could be identified, but with a very weak signal (data not shown). DBL1x-2× (3D7) and DBL3x-4ε (FCR3) could be produced in *Pichia pastoris* with very low yield (estimated yield less than 5 mg/L) even after 80 h of expression (Supplementary Figure 1).

Expression in *Lactococcus lactis* could only be assessed for DBL3x-4ε (FCR3). Indeed, only one positive clone was identified for the DBL3x-4ε (FCR3), but the protein was not detectable in bacterial lysates indicative of a very low or no expression in this system (data not shown). Despite multiple attempts, we did not succeed in sub-cloning the synthetic ORF of the other constructs in the expression vector, suggesting that these proteins are toxics for the bacteria as the cloning organism is the expression host.

**Fig. 4:**
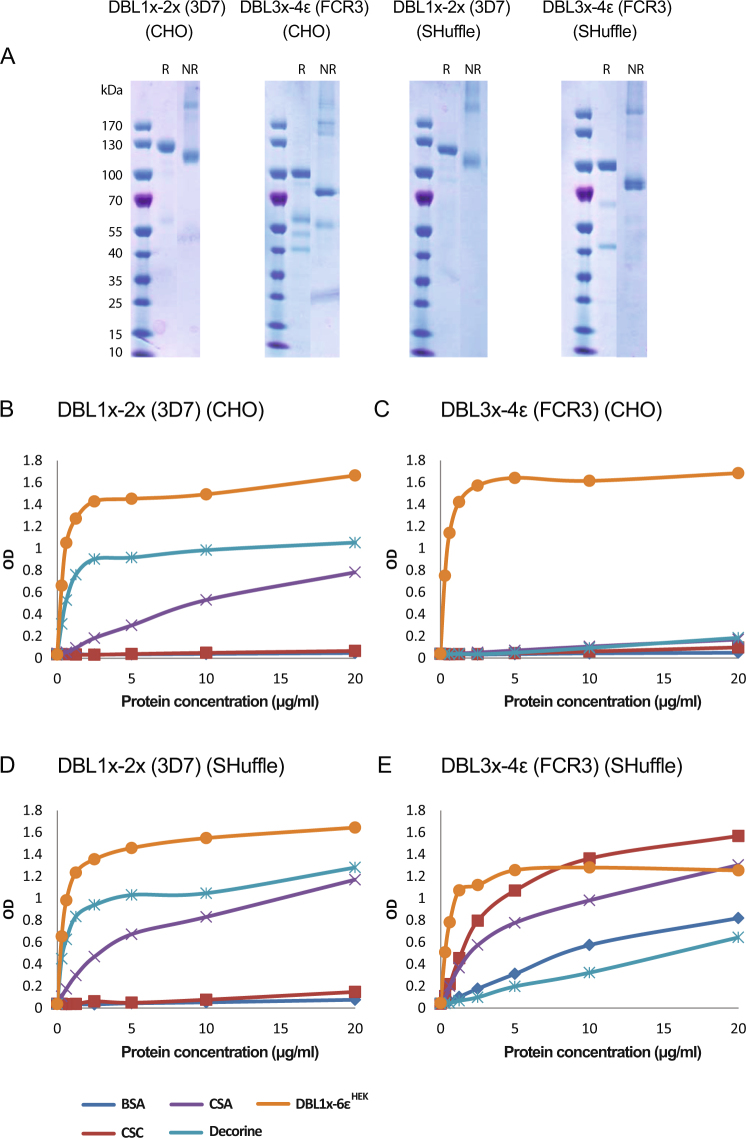
Characterization of DBL1x-2× and DBL3x-4ε expressed in either CHO cells (CHO) or in SHuffle bacteria (SHuffle). **a** Gel electrophoresis analysis of the purified recombinant proteins in reducing (R) or non-reducing (NR) conditions. **b**–**e** ELISA-based direct binding assay was performed to identify the specificity of **b** DBL1x-2× (3D7) (CHO), **c** DBL3x-4ε (FCR3) (CHO), **d** DBL1x-2× (3D7) (SHuffle), **e** DBL3x-4ε (FCR3) (SHuffle) to different sulfated glycosaminoglycans. Increasing concentrations of recombinant proteins at serial dilutions of 0.31–20 µg/mL were added to wells previously coated with BSA, CSA, and CSC glycosaminoglycans or with the CSA-carrying proteoglycan decorin. The CSA-binding recombinant protein 3D7 DBL1x-6ε^HEK^ was used as a functional control

Taken together, these results highlight that among the four different heterologous expression systems tested, CHO cells and *E. coli* SHuffle bacteria are the most suitable for producing sufficient amounts of VAR2CSA-based recombinant proteins to allow pre-clinical testing and further transition to vaccine development and notably GMP production. Out of the five recombinant proteins produced in this multisystem expression study we have down-selected the better expressed DBL1x-2× (3D7) and DBL3x-4ε (FCR3) for further assessment/development as potential vaccine candidates. DBL1x-2× (3D7) and DBL3x-4ε (FCR3) were therefore produced at larger scale in both the CHO cells (3.6 L culture supernatant) and *E. coli* SHuffle bacteria (20 L production) systems and purified by Ni-affinity followed by gel filtration chromatography. A gel electrophoresis analysis profile of the purified proteins is displayed in Fig. 4a. After purification, 7.5 mg of DBL1x-2× (3D7) and 19 mg of DBL3x-4ε (FCR3) produced in CHO cells as well as 11 mg of DBL1x-2× (3D7) and 8 mg of DBL3x-4ε (FCR3) produced in *E. coli* SHuffle were recovered. Using capillary electrophoresis, it was estimated that all the proteins have purity above 90%. Slight multimerization could be observed in non-reducing (NR) conditions for all the proteins. For DBL3x-4ε (FCR3) produced in CHO and *E. coli* SHuffle, slight degradation products in reducing conditions (R) were also observed, indicative of nicked proteins structurally maintained by disulfide bonds.

### Adhesive properties of down-selected vaccine candidates

The adhesion properties of DBL1x-2× (3D7) and DBL3x-4ε (FCR3) expressed either in CHO cells or in SHuffle bacteria were tested by direct ELISA using plates coated with different sulfated glycosaminoglycans (GAG); including CSA, CSC, and decorin, a proteoglycan exhibiting CSA chains. The full-length extracellular region of VAR2CSA (3D7) produced in HEK293 cells, DBL1x-6ε^HEK^, which was previously well characterized for its functional integrity was used as a functional control for binding to decorin in this set of experiments.^18^

As expected, DBL3x-4ε (FCR3) expressed in CHO cells did not display any binding to the different sulfated glycosaminoglycans tested (Fig. 4c). However, DBL3x-4ε (SHuffle) bound to purified CSA and CSC as well as to a lower extent to decorin and BSA indicative of a non-specific binding (Fig. 4e). In contrary, DBL1x-2× expressed in either SHuffle or CHO strongly bound to decorin and purified CSA but did not bind to CSC (Fig. 4b–d) demonstrating that these recombinant proteins retained a similar specificity compared to DBL1x-6ε^HEK^ but also to CSA-binding IEs.^18^ These results designate the DBL1x-2× (3D7) constructs, expressed in either CHO cells or SHuffle bacteria, as a potentially functional vaccine candidate.

### Immunogenicity of down-selected vaccine candidates

In order to assess the immunogenicity of each down-selected antigens (DBL1x-2× (3D7) and DBL3x-4ε (FCR3)) in small animals, Wistar rats were immunized with the different constructs either in physiological buffer (no adjuvant) or in combination with a panel of adjuvants approved for human use; alhydrogel, GLA-SE, AbISCO®-100, and also Freund’s adjuvant. The immunization scheme used for a given adjuvant is depicted in Supplementary Figure 2. Blood samples were collected at regular time points to evaluate the immunogenicity of the proteins/adjuvants combinations.

Relatively high antibody titers (IgG) were already observed within seven days after the second immunizations (day 21) with DBL1x-2× (3D7) and DBL3x-4ε (FCR3) produced in both the CHO cell and the SHuffle bacteria systems (Table 1). The proteins adjuvanted with Freund’s adjuvant generated a humoral response of higher amplitude as compared to those supplemented with Alhydrogel or GLA-SE. The antibody titers relative to each combination immunizing antigen/adjuvant tend to be higher when the recombinant proteins were produced in bacteria. When produced in the same expression system and combined with a similar adjuvant, DBL3x-4ε (FCR3) seemed, in most cases, slightly more immunogenic than DBL1x-2× (3D7). Notably, non-negligible IgG responses towards DBL1x-2× (3D7) and DBL3x-4ε (FCR3) were detected in absence of adjuvant. Following the third immunization, at day 42, antibody titer values increased but present a similar trend to those observed in samples collected at day 21. Seven days after the last injections (day 63), DBL1x-2× (3D7) and DBL3x-4ε (FCR3) in combination with Freund’s adjuvant generated higher antibody titers as compared to the other adjuvants or to physiological buffer.

**Table 1:** Antibody titers (IgG)

Bleeds								
Mean titers (standard deviation)								
	Proteins	Adjuvants	D0	D21	D35	D42	D63	D65
CHO cells	DBL1x-2×	Freund’s	<50	6017 (7918)	ND	13,000 (11,790)	29,333 (35,275)	25,333 (34,385)
	(3D7)	Alhydrogel	<50	4000 (1000)	ND	6667 (1528)	6333 (2082)	3667 (577)
		GLA-SE	<50	6333 (3055)	ND	8000 (3464)	9000 (5568)	6667 (4726)
		Abisco	<50	ND	18,333 (11,719)	ND	14,000 (7211)	8667 (4933)
		No adjuvant	<50	1467 (1343)	ND	3750^a^ (3182)	2000^a^ (1414)	2000^a^ (1414)
	DBL3x-4ε	Freund’s	<50	10,217 (17,135)	ND	13,000^a^ (11,314)	43,500^a^ (10607)	38,500^a^ (6364)
	(FCR3)	Alhydrogel	<50	6017 (6535)	ND	6333 (3512)	5333 (2517)	3333 (1155)
		GLA-SE	<50	900 (173)	ND	11,667 (2309)	15,667 (10,017)	12,333 (8505)
		Abisco	<50	ND	6333 (1155)	ND	15,000 (9539)	15,000 (12,124)
		No adjuvant	<50	67 (29)	ND	1133 (321)	2000 (1000)	1833 (1041)
*E. coli*	DBL1x-2x	Freund’s	<50	43,000 (10536)	ND	31,667 (11,240)	21333 (11372)	15,000 (11,269)
	(3D7)	Alhydrogel	<50	14,000 (8544)	ND	11,000 (10,583)	6333 (4163)	5000 (2646)
		GLA-SE	<50	11333 (8021)	ND	14,667 (8386)	12,667 (7024)	11,000 (8544)
		Abisco	<50	ND	15,667 (13,868)	ND	7000 (4583)	6000 (5000)
		No adjuvant	<50	333 (491)	ND	1667 (2031)	1400 (1400)	1333 (1457)
	DBL3x-4ε	Freund’s	<50	69,667 (71,842)	ND	115,000 (142,902)	109,333 (139,317)	84,333 (108,969)
	(FCR3)	Alhydrogel	<50	26,667 (32,470)	ND	16,000 (11,269)	12,667 (6658)	10,333 (4933)
		GLA-SE	<50	5633 (4099)	ND	15,667 (9074)	13,667 (10693)	8333 (7506)
		Abisco	<50	ND	8733 (11,645)	ND	33,333 (26633)	21,333 (13,650)
		No adjuvant	<50	200 (173)	ND	4333 (577)	3000 (1732)	2000 (1000)

^a^Indicates that the mean titers result from only two animals

After the course of the immunization process, GLA-SE and AbISCO®‐100 appeared to be more potent adjuvants than Alhydrogel, giving rise to higher IgG titers towards the recombinant proteins of interest. Although the rats only received three injections of the proteins formulated with AbISCO®-100, antibody titers at days 63 and 65 were most of the time higher than for rats that received four injections of the proteins adjuvanted with GLA-SE or Alhydrogel.

Functional characterization of antibodies generated in small animal immunization with the down-selected vaccine candidates

Each down-selected recombinant proteins (DBL1x-2× and DBL3x-4ε), produced in the CHO cells or the SHuffle bacteria systems were tested for their capabilities to generate in rats antibodies which are able (i) to cross-recognize infected erythrocytes expressing different VAR2CSA variants and (ii) to cross-inhibit the binding of infected erythrocytes expressing different polymorphisms of VAR2CSA to CSA.

**Fig. 5:**
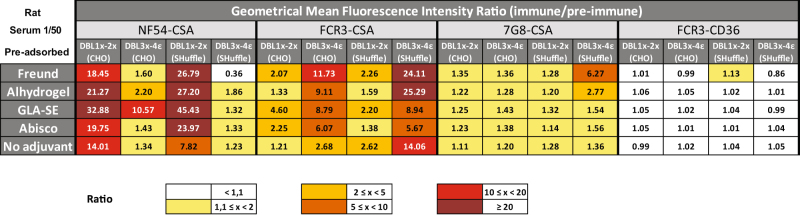
Immune recognition of erythrocytes infected by different parasite strains (NF54, FCR3, and 7G8) selected for different adhesive phenotypes (CSA and CD36) by vaccination-induced antibodies directed towards VAR2CSA-based constructs. Infected erythrocytes were incubated with rat sera samples diluted 1:50 in PBS 1% BSA. Erythrocyte-bound IgGs were detected using a rat IgG specific antibody PE-conjugated. Cells were then subjected to flow cytometry analysis. Results are expressed as the fold-increase in geometrical mean fluorescence intensity obtained with immune (Day 63) sera compared to non-immune sera. For each condition, the fold-increase values obtained for the diluted sera represent the pool of three rat sera samples

Day 63 sera samples (diluted 1/50) resulting from different immunization regimens were co-incubated with NF54-CSA, FCR3-CSA, or 7G8-CSA IEs selected for binding to CSA and expressing VAR2CSA on their surface or to CD36-binding IEs (FCR3-CD36). NF54-CSA IEs expressed at their surface the autologous VAR2CSA variant from which the DBL1x-2× (3D7) protein is derived, whereas FCR3-CSA IEs expressed the VAR2CSA variant corresponding to the DBL3x-4ε (FCR3) protein. Flow cytometry assessment of membrane-bound IgG revealed that, as compare to preimmune sera, rats immunized with DBL1x-2× (3D7) expressed in either CHO cells or SHuffle bacteria were able to generate antibodies able to strongly recognize native VAR2CSA from the autologous parasite strain NF54-CSA and to cross-react with cell-surfaced exposed VAR2CSA from FCR3-CSA parasites and to a lower extent from 7G8-CSA parasites (Fig. 5). Such recognition tended to be more pronounced when animals were immunized with antigens supplemented with Freund’s adjuvant, alhydrogel or GLA-SE as compared to AbISCO®‐100 or physiological buffer. Samples from rats immunized with DBL3x-4ε (FCR3) also showed a good reactivity towards the autologous VAR2CSA variant expressed at the surface of erythrocytes infected with FCR3 as well as towards native heterologous VAR2CSA protein from NF54 parasites. As for DBL1x-2× (3D7) samples, cross-recognition of 7G8-CSA IEs was weaker than the one observed for NF54-CSA. Notably, the autologous recognition of IEs (DBL1x-2× vs. NF54-CSA and DBL3x-4ε versus FCR3-CSA) tend to be more pronounced when IEs where incubated with sera from animals immunized with *E. coli* expressed recombinant proteins as compared to CHO cells produced constructs. None of the samples significantly reacted to FCR3-CD36. These results demonstrate that both vaccine candidates DBL1x-2× (3D7) and DBL3x-4ε (FCR3) are potent to generate in rats antibodies that are able to cross-react with native VAR2CSA from different parasite strains regardless the expression system they are expressed in. A better overall recognition of VAR2CSA expressing IEs was observed with samples from animals which receive an immunizing cocktail comprising the Freund’s adjuvant, alhydrogel, or GLA-SE.

**Fig. 6:**
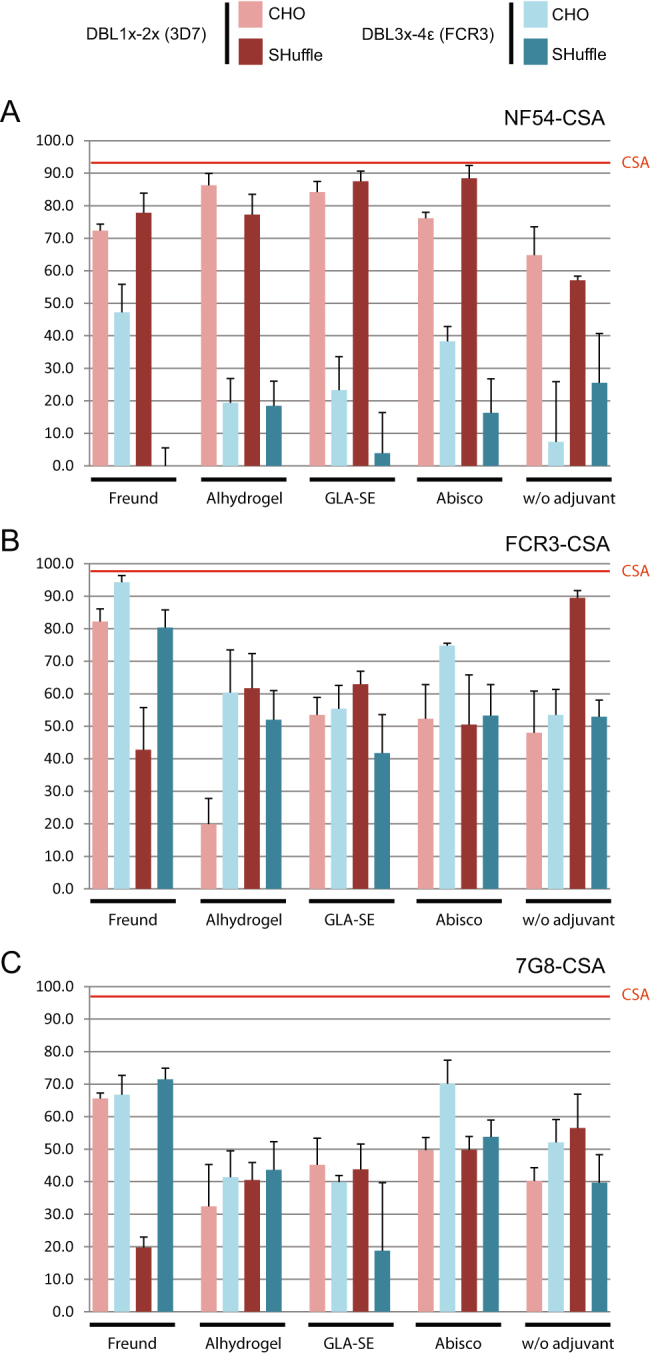
Inhibition of infected erythrocytes binding to CSA by vaccination-induced antibodies directed towards VAR2CSA-based constructs. **a** NF54-CSA, **b** FCR3-CSA, **c** 7G8-CSA IEs were pre-incubated with rat sera samples (pool of three rats for each condition) diluted 1:10 and then plated on CSA-coated plates. CSA-binding inhibition was assessed by relative quantification of IEs remaining bound to the plate surface. For a given condition, the percentage of inhibition was calculated as follow: %_inhibtion_ = 100 − (OD_immune_/OD_preimmune_) × 100. The displayed results represent three independent experiments. The red lines correspond to the % of inhibition obtained with 1 mg/mL of soluble CSA

The capabilities of vaccination-induced antibodies directed towards the down-selected VAR2CSA-based candidates to inhibit the interaction of VAR2CSA expressing IEs with CSA were then assessed in a high throughput 96-well assay. Day 63 sera samples (diluted 1/10) were co-incubated with NF54-CSA, FCR3-CSA or 7G8-CSA IEs. Plasma samples from animals immunized with DBL1x-2× (3D7) expressed in either CHO cells or SHuffle bacteria were able to strongly inhibit the interaction of NF54 IEs to CSA (Fig. 6a). The inhibition was more pronounced (>70%) when animals where immunized with DBL1x-2× (3D7) combined with an adjuvant as compared to physiological buffer. A good cross-inhibition was also observed for FCR3-CSA IEs (Fig. 6b) and to a lower extent for 7G8-CSA IEs (Fig. 6c). Curiously, rat sera from Freund’s adjuvanted DBL1-2 expressed in SHuffle bacteria had less inhibitory properties on FCR3-CSA and 7G8-CSA than when formulated with the other adjuvants and even without adjuvant (Fig. 6b, c). In contrast, day 63 sera from animals receiving DBL3x-4ε (FCR3) as an antigen were able to strongly inhibit the interaction of erythrocyte infected by the autologous strain FCR3 to CSA (Fig. 6b) but poorly inhibit NF54-CSA IEs adhesion (Fig. 6a). Similar inhibition properties on 7G8-CSA IEs were observed with sera from animals that received the DBL3x-4ε (FCR3) or DBL1x-2× (3D7) antigens (Fig. 6c). Taken together sera from animals immunized with the DBL1x-2× (3D7) antigens expressed in both systems tend to have better cross-inhibitory properties than the sera from rats immunized with the DBL3x-4ε (FCR3) proteins.

## Discussion

Mammalian, yeast and insect cells are suitable platforms to express complex multi-disulfide-bonded proteins. Nevertheless, these systems face some limitations, notably their elevated costs as well as a relatively slow production process. Insect cells, for instance, require long lasting set up procedures with constraining production scale up. Furthermore, the system reproducibility is relatively instable since it mostly depends on the state of the cells at a given time and on the generation of very variable viral stocks. It was therefore decided in this multi-system feasibility study to exclusively focus on one mammalian system, the CHO cells; one fungus, *Pichia pastoris*; and on two prokaryotic platforms, *Lactococus lactis* and *E. coli*.

Our results demonstrated that the CHO cells and *E. coli* expression systems were the most suitable to produce large amounts of these high molecular weight cysteine-rich recombinant proteins allowing further efficient purification steps and downstream testing. DBL1x-2× (3D7) and DBL3x-4ε (FCR3) expression levels in both CHO cells and *E. coli* SHuffle reached very high values with production yields ranging from 67 to 133 mg/L of culture. Remarkably, SHuffle bacteria were more potent to produce these constructs in large quantities as compared to BL21 or HMS174. Bacterial disulfide-bound formation of proteins requires their translocation to the periplasmic compartment of the cell via an N-terminal signal peptide.^25^ Expression of complex multi-disulfide-bonded recombinant proteins in standard *E. coli*-based systems is therefore usually leading to low production yields. Technological advances permitted to overcome this constraint by generating mutant *E. coli* strains capable of promoting disulfide bound formation in the cytoplasm leading to more efficient folding of recombinant proteins, with increased production yields.^26,27^ The SHuffle bacteria, based on the trxB *gor* suppressor strain SMG96 were engineered to achieve such purposes by diminishing the reductive pathway in the cell cytoplasm.^28^ Our results are therefore in line with the previously described benefits of such technologies.

Functional analysis of DBL1x-2× (3D7) and DBL3x-4ε (FCR3) expressed in either CHO cells or *E. coli* SHuffle revealed a disparity for the recombinant proteins in terms of binding specificity and affinity to CSA. DBL1x-2× (3D7) produced in both expression systems presented similar CSA-binding specificity and affinity than the full-length DBL1x-6ε (3D7) expressed in HEK293 cells.^18^ This indicates that DBL1x-2× (3D7), expressed in either CHO cells or SHuffle bacteria, most likely harbor structural features comprising a fully functional CSA-binding site similar to the one of full-length protein. Such constructs are therefore interesting candidates to generate antibodies that would block the adhesion of IEs to the placenta by targeting residues directly involved in CSA-binding.

DBL3x-4ε (FCR3) does not possess the CSA-binding site, which lays in the N-terminal part of the VAR2CSA protein (ID1-ID2a sequence).^19^ The rationale behind the assessment of such a construct as a PM vaccine candidate relies on the higher degree of conservation of DBL4ε domain among various strains^29^ and on the fact that CSA-binding could be affected by antibody binding to epitopes in close proximity of the site, thus restraining CSA accessibility by steric hindrance. When expressed in CHO cells, DBL3x-4ε (FCR3) did not bind to CSA. However, when expressed in *E. coli* SHuffle bacteria, DBL3x-4ε (FCR3) revealed a strong binding to CSA but also to another type of chondroitin sulfate, CSC as well as a non-negligible binding to BSA. DBL3x-4ε (FCR3) being devoid of the CSA-binding site, these results are indicative of a non-specific binding that could arise from a miss-folding of the protein in this expression system, resulting in the exposition of positively charged residues normally hidden.

Taken together, these results allowed us to down-select two proteins (DBL1x-2× (3D7) and DBL3x-4ε (FCR3)) and two expression systems (CHO and *E. coli* SHuffle bacteria) for further vaccine candidate pre-clinical testing.

Clinical protection against the severe symptoms associated with placental malaria have been linked to the presence of antibodies that are able to recognize the surface of VAR2CSA expressing IEs and to inhibit the interaction of said IEs with the syncytiotrophoblastic lining of the placenta.^6–8^ In order to evaluate the capabilities of the down-selected vaccine candidates to generate a potent immune response in small animals, rats were immunized with DBL1x-2× or DBL3x-4ε expressed in either CHO cells or *E. coli*, in combination with different adjuvants. It was notable that the four recombinant proteins used in this pre-clinical study were very immunogenic, high serological levels of anti-VAR2CSA antibodies being already reached 7 days after the third injection of the immunizing scheme, even without adjuvant. Freund’s adjuvant was the most potent at generating high antibody titers. Freund’s adjuvant is to date the gold standard for achieving hyper-immunization^30^ in small animals against a larger variety of antigens and was therefore included in this work as a reference. Due to its proven toxicity, Freund’s adjuvant is forbidden for human use. Even though GLA-SE and AbISCO®‐100 appeared to be more efficient than Alhydrogel at generating IgG against VAR2CSA-derived recombinant proteins, we observed a disparity between the antibody titers obtained by ELISA on the recombinant proteins and the recognition of native VAR2CSA expressed at the surface of CSA binding IEs. Indeed, the best immune recognition of VAR2CSA expressing IEs was mostly observed when animals were immunized with recombinant proteins adjuvanted with Alhydrogel or GLA-SE, two adjuvants designed for human use.

Sera from animals immunized with DBL1x-2× (3D7) expressed in either CHO cells or SHuffle bacteria were able to strongly inhibit the interaction of the autologous strain NF54 to CSA and to a lower extent for 7G8-CSA and FCR3-CSA IEs. In contrast, plasma samples from DBL3x-4ε (FCR3) immunized animals were also able to strongly inhibit the interaction of the autologous FCR3-CSA IEs to CSA but more modestly inhibit the binding of NF54-CSA IEs to CSA.

Although the DBL3x-4ε overall conservation in VAR2CSA sequence is much higher than DBL1x-2× ^29^ (Table S3), this result seems to indicate that DBL3x-4ε has less capacity to generate inhibitory antibodies certainly due to the fact that the CSA binding site is localized with the ID1-ID2a^19^ region and that these inhibitory antibodies are not directed against the CSA binding pocket but instead could affect CSA binding by steric hindrance.

Indeed, small angle x-ray scattering studies revealed a compact organization of the full-length extracellular region of VAR2CSA,^18^ the CSA-binding site being most likely formed by a higher-ordered structure involving more than a single DBL.^16,17^ This data highlights the potential importance of conformational epitopes in both the recognition of native VAR2CSA as well as in the blocking of the CSA-binding pocket. The constructs boundaries, as they were chosen in this study, may result in the exposure of artificial immunogenic epitopes in the recombinant proteins, which are not present in the native form of VAR2CSA. Since such antibodies might not be of real relevance in terms of protection, the VAR2CSA immune recognition assessment performed by flow cytometry would be more informative than the ELISA with regards to clinical aspects.

Autologous strain recognition was of higher amplitude when recombinant proteins used for immunizations were expressed in the *E. coli* system. These observations may reflect a better folding of the VAR2CSA-derived vaccine candidates in SHuffle bacteria than in CHO cells and are thus in line with the disparity observed during the functional analysis of the recombinant proteins expressed in different systems.

Our data also revealed that infected erythrocyte surface reactivity does not completely align with binding inhibition. This is particularly evident for erythrocytes infected with the 7G8 parasite strain. Indeed, the observed cross-reactivity is relatively low for most of the samples on 7G8-CSA IEs (Fig. 5), whereas the same plasmas still demonstrate a decent capability to inhibit CSA-binding of those parasites (Fig. 6). These results imply that CSA-binding inhibition could be induced by limited amounts of inhibitory antibodies, sometimes barely detectable at the infected erythrocyte surface by flow-cytometry. Consequently, VAR2CSA seems to harbor a limited number of accessible conserved epitopes relevant for CSA-inhibition by immunoglobulins. Since the presence of antibodies that are able to recognize the surface of VAR2CSA expressing IEs has been linked to protection against the severe clinical outcomes of PM,^6,8^ IEs surface cross-reactivity appears as a desirable facet when developing a PM vaccine but our work tend to suggest that CSA-adhesion blocking of infected erythrocytes, also linked to protection, might be the key element to evaluate in pre-clinical study in order to decide whether the candidate should be taken forward or not. It is nevertheless still unclear whether inhibition of IEs to CSA alone is sufficient to confer full protection against the dire clinical outcomes of PM or if some additional immune mechanisms must be elicited. Some recent studies call for developing new assays to measure the ability of vaccines to promote opsonic phagocytosis and activate the classical pathway of the complement system,^31^ two immune effectors that could arise from IEs recognition by antibodies.

In this multi-system feasibility study, we have decided to exclusively focus on expression systems already used for commercial manufacturing of biological drugs (CHO cells; one fungus, *Pichia pastoris*; and on two prokaryotic platforms, *Lactococus lactis* and *E. coli*). The other placental malaria development project (PAMCPH/PlacMalVac), has opted for the *Drosophila* Schneider-2 cell (S2) expression-system to produce the ID1-DBL2X-ID2a, VAR2CSA (FCR3)-based vaccine candidate.^21^

In comparison to other expression systems, protein expression in bacterial systems presents great advantages in the context of vaccine production where time and cost are key factors from the developmental phase to the potential market release. Indeed, bacterial expression platforms are relatively easy to establish and to scale up allowing a straightforward transition to current GMP manufacturing. Furthermore, this type of system is cost effective and most often offers very good production yield suitable with vaccine testing in clinical trials and with potential marketing.

In this work, DBL1x-2× expressed in *E. coli* SHuffle bacteria stood up as the best antigen to be transitioned to further clinical development in order to protect future pregnant women living in malaria endemic areas against the severe clinical outcomes of placental malaria. This work paved the way for the clinical development of an easily scalable low cost effective vaccine, currently being tested in a Phase Ia/Ib Clinical trial (ClinicalTrials.gov Identifier: NCT02658253), that could prevent placental malaria and then save an estimated 10,000 maternal and 200,000 infant deaths annually.^3–5^

## Methods

### Multi-system protein expression

#### Chinese Hamster Ovary (CHO) cells

Synthetic genes were designed for DBL1x-6ε (3D7), DBL1x-3× (3D7), INT1-DBL3x (3D7), DBL1x-2× (3D7) (PFL0030c) with optimization according to *Homo sapiens* codon usage.^18^ For DBL3x-4ε (FCR3) (IT4var04), a synthetic gene was designed with optimization according to *Cricetulus griseus* codon usage. The mean difference between *Homo sapiens* and *Cricetulus griseus* codon usage is 4.16%, thus not significant. Since *P. falciparum* proteins are not N-glycosylated, all serines or threonines at potential N-glycosylation sites were substituted by alanine in the synthetic genes. The DNA sequences encoding the proteins were cloned into a proprietary expression plasmid (GTP Technology, Toulouse, France) in fusion with the mouse Ig kappa signal peptide at the 5′ end and six histidines tag with a linker region (GGSSHHHHHH) at the 3′ end. Each expression plasmid was transfected into CHO-S cells to generate stable recombinant cell lines. A master cell bank was established and stored in liquid nitrogen. Following a freeze-thaw cycle, the recombinant cell lines were cultured in a synthetic medium to produce the protein of interests according to an optimized process (CHO-Xpress®; GTP Technology, Toulouse, France). All VAR2CSA-variants were expressed in the culture supernatant. The culture supernatants were harvested every day up to 7 days post-transfection and stored at −20 °C for further analysis. Proteins of interest were purified by Ni-affinity on Ni-Sepharose 6FF columns (GE Healthcare) and then subjected to gel filtration chromatography in 50 mM Tris; 200 mM NaCl on a Superdex 200 column (GE Healthcare).

#### Pichia pastoris

Synthetic genes were designed for DBL1x-2× (3D7), DBL1x-3× (3D7), INT1-DBL3x (3D7) (PFL0030c), and DBL3x-4ε (FCR3) (IT4var04) with optimization according to *Pichia pastoris* codon usage. All serines or threonines at potential N-glycosylation sites were substituted by alanine in the synthetic genes. The DNA sequences encoding each protein were cloned into the pPIC9K expression plasmid (Thermo Fisher Scientific) in fusion with the alpha factor signal peptide at the 5′ end and six histidines-Flag tag with a linker region (GGSSHHHHHHDYKDDDDK) at the 3′ end. Each expression vector was linearized by single digestion *Sac*I, in the AOX1 promoter, and transfected into GS115 cells (Thermo Fisher Scientific) to generate stable recombinant His + Mut + clones, with G418 and His selection on agar medium. A screening of 250 recombinant clones was done by Colony Lift Immunoassay to select the best producer clones. The Dot-Blot revelation was performed using an anti-Flag®M2 antibody (Sigma). For each recombinant protein producing strain, a glycerol stock was stored at −80 °C. After thawing, selected clones expressing the protein of interest were cultured in flasks by standard fed batch mode. Samples of supernatants were harvested and stored at −20 °C every 24 h, for 4 days.

#### Lactococcus lactis

Synthetic genes were designed for DBL1x-2× (3D7), DBL1x-3× (3D7), INT1-DBL3x (3D7) (PFL0030c) and DBL3x-4ε (FCR3) (IT4var04) with optimization according to *Lactococcus lactis* codon usage. The DNA sequences encoding each protein were cloned into a proprietary expression plasmid (GTP Technology, Toulouse, France) in fusion with the Exp4 signal peptide at the 5′ end and six histidine tag with a linker region (GGSSHHHHHH) at the 3′ end. MG1363-htra (LA17) bacteria strain was transformed with the expression plasmids and selected on agar medium with chloramphenicol. For each recombinant protein producing clones, a glycerol stock was established and stored at −80 °C. After thawing, selected clones expressing the protein of interest were cultured in flasks by batch mode in six different conditions, varying the inducer concentration, the pH of the culture and the conditions of induction (Lact-Express® process; GTP Technology, Toulouse, France). Samples of culture supernatants were harvested at four induction times (0, 1, 3, and 5 h) and stored at −20 °C.

#### Escherichia coli

Synthetic genes were designed for DBL1x-2× (3D7), DBL1x-3× (3D7), INT1-DBL3x (3D7) (PFL0030c) and DBL3x-4ε (FCR3) (IT4var04) with optimization according to *Escherichia coli* codon usage. The DNA sequences encoding each protein were cloned into a proprietary expression plasmid (GTP Technology, Toulouse, France) in fusion with six histidines tag with a linker region (GGSSHHHHHH) at the 3′ end. The expression vector optimized for industrial transfer included the following features: a tac expression promoter used for a high and regulated expression, inducible with IPTG^32^ and a kanamycin resistance open reading frame (ORF), compatible with GMP production. Three different *E. coli* strains (SHuffle; New England Biolabs, BL21; New England Biolabs and HMS174; Novagen) were transformed with each expression plasmid and selected on agar medium with kanamycin. For each selected clones, a glycerol stock was established and stored at −80 °C. After thawing, selected clones expressing the protein of interest were cultured in flasks by batch mode in 6 different conditions, varying the type of medium (Glycerol/Yeast (GY) extract or the auto-inducible medium Glucose/Glycerol/Lactose/Yeast extract/Peptone (GGLYP20)), the growth, induction temperature and the induction duration (Table S2). Cell samples were harvested and stored at −20 °C before and after induction. Proteins of interest were purified by Ni-affinity on Ni-Sepharose 6FF columns (GE Healthcare) and then subjected to gel filtration chromatography in 50 mM Tris; 200 mM NaCl on a Superdex 200 column (GE Healthcare).

### Animal immunization

All animal vaccination experiments were executed in strict accordance with good animal practices, following the EU animal welfare legislation and after approval of the Biotem and INSERM ethical committees. Every effort was made to minimize suffering. Immunization of Wistar rats with the recombinant proteins were performed by Biotem. Wistar rats (6 weeks old) were randomly divided into groups, with three animals each. The size of the groups was choosen in order to be able to distinguish differences in humoral immune responses. Rats received 3 or 4 injections of DBL1x-2× (3D7) and DBL3x-4ε (FCR3) expressed either in *E. coli* or CHO cells in combination with a panel of four different adjuvants (Freund, Alhydrogel, IDRI GLA-SE or Abisco®-100) or without adjuvant following the immunization procedures described in Supplementary Figure 2. These groups were not blinded to the investigators. Alhydrogel® adjuvant 2% [alhydrogel] (Brenntag, Denmark) is an aluminum hydroxide wet gel suspension. Glucopyranosyl Lipid Adjuvant‐Stable Emulsion [GLA‐SE] (Infectious Disease Research Institute, USA) is a synthetic Toll‐like receptor (TLR‐4) agonist formulation^33,34^ and AbISCO®‐100 (Isconova AB, Sweden) is a saponin‐based adjuvant.^35^ Rats received either 10 µg of TLR-4 agonist (GLA-SE), 250 µg of Aluminum (Alhydrogel®) or 30 µg of AbISCO®‐100 at each injection.

### Data availability

Materials described in the manuscript, including all relevant raw data, will be freely available to any scientist wishing to use them for non-commercial purposes, without breaching participant confidentiality.

## Electronic supplementary material

Supplementary information
