# Supplementary material for: Down-selection of the VAR2CSA DBL1-2 expressed in E. coli as a lead antigen for placental malaria vaccine development
Source: NPJ Vaccines. 2018 Jul 17;3:28. doi: 10.1038/s41541-018-0064-6 (PMC6050242; doi:10.1038/s41541-018-0064-6)
Supplement: Supplementary file 1 — Supplementary information [file 41541_2018_64_MOESM1_ESM.pdf]

SUPPLEMENTAL MATERIALS AND METHODS

Western blotting

Soluble protein extracts were loaded on 4–12% Bis-Tris SDS-PAGE gels (Invitrogen) under reducing or non-reducing conditions and subjected to electrophoresis. Proteins were then transferred onto polyvinylidene difluoride (PVDF) membranes. Membranes were blocked with a casein-containing buffer (Qiagen) for 1h at room temperature (RT). An HRP-conjugated anti-His antibody (ref 34660; Qiagen) was added to the membranes at 1:2,000 in blocking buffer and incubated for 1h at RT. Membranes were then washed twice with TBST and once with TBS for 10 min before revelation with SuperSignal West Pico Chemiluminescent Substrate (Thermo Scientific). All blots derive from the same experiment and were processed in parallel.

Binding of VAR2CSA-derived proteins to glycosaminoglycans

Ultra-high binding flat bottom microtiter plates (Immunolon 4HBX 3855) were coated overnight at 4 °C with different glycosaminoglycans (100 µL per well): 50 µg/mL for chondroitin sulfate A (CSA) (Sigma, C8529), chondroitin sulfate C (CSC) (Seikagaku, 400670) and 5 µg/mL for the decorin, a CSA chains carrying protein (Sigma, D8428) diluted in phosphate buffered saline (PBS) pH 7.2 (Gibco). Plates were then washed 3 times with PBS and blocked for 1h at 37 °C with dilution buffer (PBS 1% bovine serum albumin (BSA), 0.05% Tween20). After removing the blocking solution, serial dilutions (0.3125–20 µg/mL) of each VAR2CSA-derived recombinant protein were added to the wells and incubated for 1 h at 37 °C with gentle shaking. Plates were washed three times with PBS containing 0.05% Tween 20 (PBST). One hundred microliters of anti-His HRP-conjugated antibody (Qiagen) at 1/2,000 in dilution buffer were added to each well and incubated for 1 h at 37 °C. Plates were

then washed three times with PBST and 100 µL of TMB (3,3',5,5'-tetramethylbenzidine) substrate (Biorad) were added per well. Absorbance was measured after 20 min at 655 nm on a microplate reader.

VAR2CSA-derived protein specific IgG titers

Ultra-high binding flat bottom microtiter plates (Immunolon 4HBX 3855) were coated overnight at 4°C with 1 µg/ml of immunizing antigen in PBS. Plates were then washed 3 times with PBS and blocked for 1h at 37 °C with dilution buffer (PBS 1% BSA). After blocking solution removing, rat sera serial dilutions (1/100 to $1/10^7$) were added to the wells and incubated for 1 h at 37 °C with gentle shaking. Plates were washed three times with PBS 1% BSA One hundred microliters of anti-rat IgG (Fc-specific) HRP-conjugated antibody (ref 112-035-008; Jackson Immunoresearch) at 1/4,000 in dilution buffer were added to each well and incubated for 1 h at 37 °C. Plates were then washed three times with PBST and 100 µL of TMB (3,3',5,5'-tetramethylbenzidine) substrate (Biorad) was added per well. Absorbance was measured at 655 nm on a microplate reader. After data plotting and a 4-parameter logistic regression curve fitting, the plasma dilution corresponding to 50% of the maximal OD value (sigmoid curve plateau) was regarded as the VAR2CSA-derived protein specific antibody titer.

Parasite culture

P. falciparum laboratory adapted parasite strains NF54 , FCR3 (IT4) and 7G8 were grown in O^+ human erythrocytes in RPMI 1640 medium (Gibco) containing 25 mM Hepes, 2 mM L-glutamine (Gibco) supplemented with 5% Albumax (Gibco), 5% Human serum, 0.1 mM hypoxanthine (Gibco) and 20 µg/ml gentamicin (Gibco) [32]. Parasites were routinely genotyped by PCR using MSP1/MSP2 primers [33] and tested for potential mycoplasma

contamination (LookOut Mycoplasma PCR Detection Kit, Sigma). Knob-positive infected erythrocytes were routinely selected by gelatin flotation using Plasmion (Fresenius Kabi) [34]. Erythrocytes infected with NF54, FCR3 (IT4) and 7G8 were selected for the CSA-binding phenotype by multiple panning rounds on CSA (Sigma). These selected populations are referred to as NF54-CSA, FCR3-CSA and 7G8-CSA. Erythrocytes infected with FCR3 were also selected for the CD36-binding phenotype by multiple panning rounds on recombinant CD36 (R&D systems) and are referred to as FCR3-CD36. Erythrocytes infected with trophozoite stage parasites (25h-30h post-invasion) were purified from synchronized parasite cultures (3-6% parasitemia) using a VarioMACS system coupled to CS columns (Miltenyi Biotec) as previously described [35]. *Var* gene transcriptional profiling was routinely performed on NF54-CSA and FCR3-CSA to ensure correct selection of VAR2CSA expressing infected erythrocytes as well as CSA-binding assays for 7G8-CSA.

Flow cytometry-based immune recognition assays

Purified infected erythrocytes (trophozoite parasite stage; 25h-30h post-invasion) were incubated for 15 min at 37°C in PBS (Gibco; pH 7.2) supplemented with 1% BSA Fraction V (Roche) and added to a 96-well, round bottom, microplate (200,000 IEs/well). Cells were pelleted by centrifugation at 300g for 3 min at 37°C and resuspended in 100 µl of plasma or purified IgG diluted in PBS 1% BSA. Rat plasma samples were pre-adsorbed on non-infected red blood cells prior the experiment. After an hour of incubation at 37°C, cells were washed three times with 200 µl PBS 1% BSA and were then resuspended in 100 µl PBS 1% BSA containing an anti-rat IgG PE-conjugated antibodies (ref 112-116-071; Jackson ImmunoResearch; 1/100). After 45 mins of incubation at 37°C in dark, cells were washed three times with 200 µl PBS and then fixed overnight at 4°C with 4% paraformaldehyde (PFA) (Electron Microscopy Sciences) diluted in PBS. Before flow cytometry acquisition,

cells were washed once with 200 µl PBS 1% BSA and resuspended in PBS supplemented with TO-PRO®-3 (Molecular probes) according to the manufacturer's instructions. Data acquisition was performed using BD FACSCanto™ II flow cytometer (Becton-Dickinson). Infected erythrocytes gating was performed based on their morphological features using the forward (FSC) and side (SSC) scatters. TO-PRO®-3 positive cells (parasite DNA staining) were regarded as infected. Data were then analyzed in FLOWJO 8.1 (Tree Star Inc.) software. Results were expressed as the ratio of the geometrical mean fluorescence intensities (Geo. MFI) in the PE channel of the immune samples over the respective non-immune samples.

High throughput CSA-binding inhibition assays

Inhibition of infected erythrocytes binding to CSA by plasma samples was assessed in a 96-well plate in high throughput format. The assay was performed as described previously [36]. Briefly, MaxiSorp™ high protein-binding capacity polystyrene 96 well ELISA plates (Nunc) were coated with CSA (Sigma) at 1 mg/ml; 100 µl/well or BSA (Roche) at 1%; 100 µl/well diluted in PBS and incubated overnight at 4°C. Plates were then washed three times with 200 µl RPMI 1640 supplemented with 2% Fetal Bovine Serum (FBS) (Dominique Dutcher). Purified infected erythrocytes (trophozoite parasite stage; 25h-30h post-invasion) were pre-incubated with plasma samples diluted in RPMI 2% FCS (1/50) for 1h at 37°C. Infected erythrocytes were then added into the pre-coated wells (10^6 cells; 100 µl/well) and incubated for 1h at 37°C. Plates were then washed three times with 200 µl PBS by quick inversion. Infected erythrocytes remaining attached to the surface were lysed by addition of 50 µl 3,3′,5,5′-Tetramethylbenzidine (TMB) (Biorad) followed by 30 sec vortexing on a MixMate® mixer (1000 rpm). The blue-colored reaction product resulting from the pseudo-peroxidase activity of the remaining hemoglobin contained in infected erythrocytes absorbs light at 655 nm and the corresponding O.D. can be read using an iMark™ microplate absorbance reader

(Biorad). Results were expressed as % of inhibition of the immune samples compared to the respective non-immune samples [% inhibition = 100- ($OD_{immune}/OD_{pre-immune}$)/100].

SUPPLEMENTARY FIGURE S1

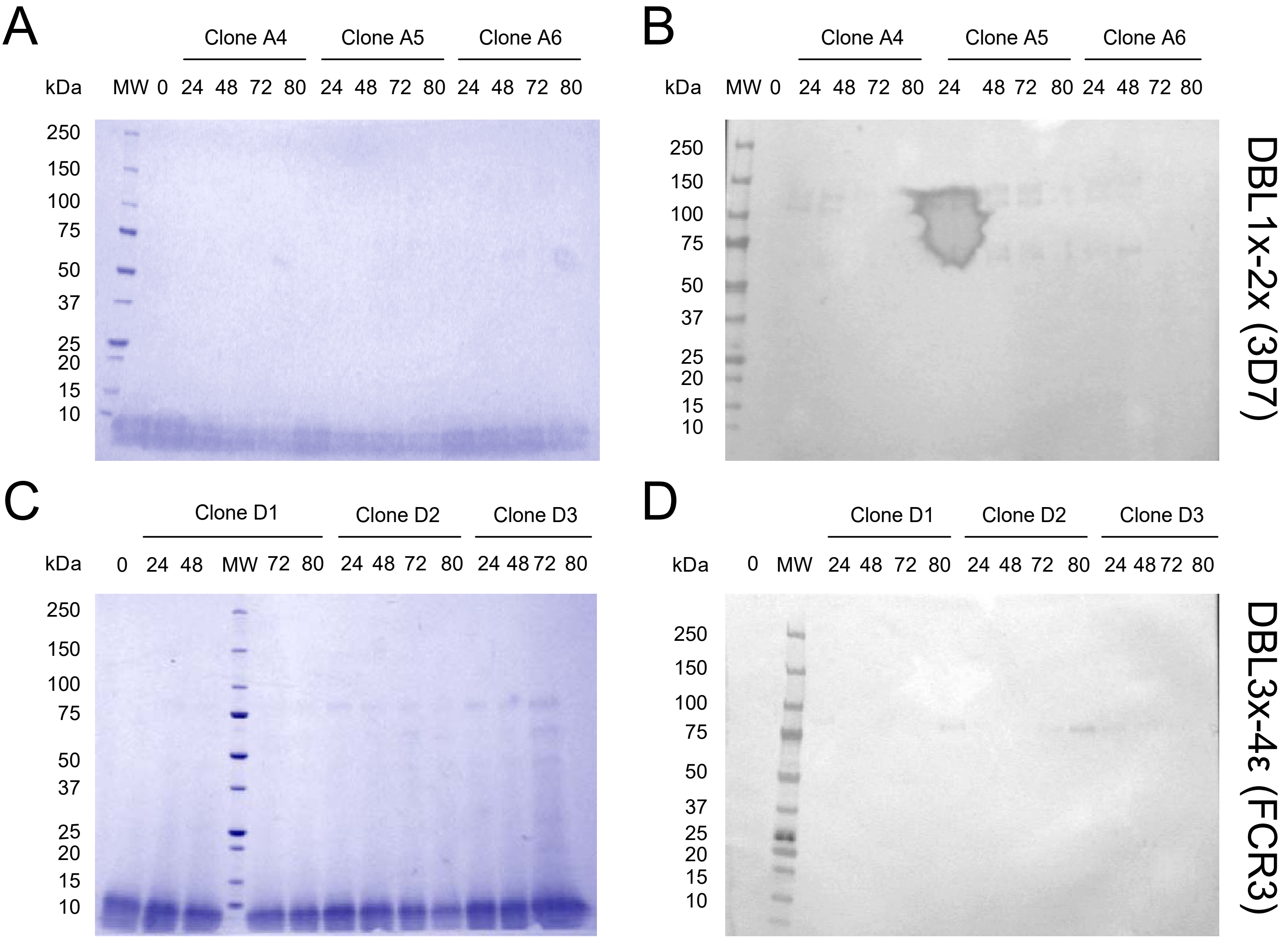

Expression analysis in *P. pastoris* system of **(A-B)** DBL1x-2x (3D7); 105 kDa and **(C-D)** DBL3x-4ε (FCR3); 86 kDa. The left panels represent the Coomassie staining of crude culture supernatants (3 clones for each construct) after gel electrophoresis in reducing and non-reducing conditions at the time of induction (0) and at different harvesting times (24h to 80h). The right panels display the western blot analysis of protein production using an anti-His antibody. MW: Molecular Weight ladder.

SUPPLEMENTARY FIGURE S2

A

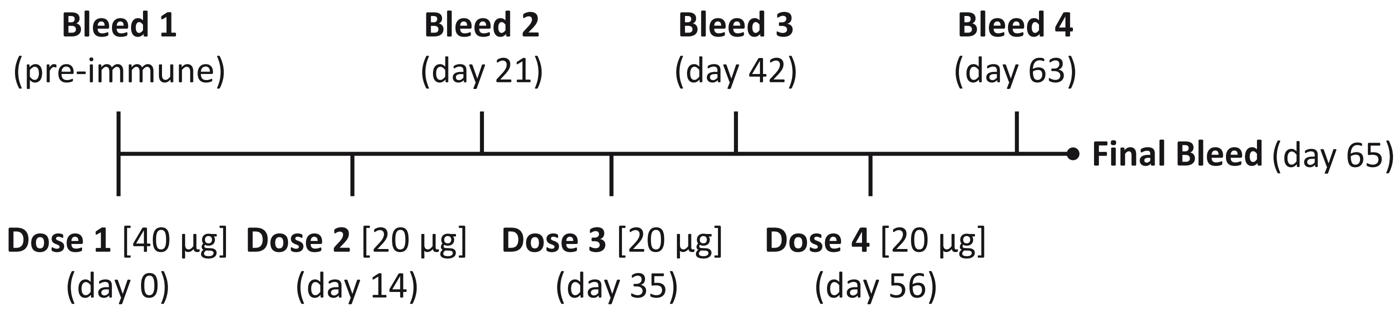

B

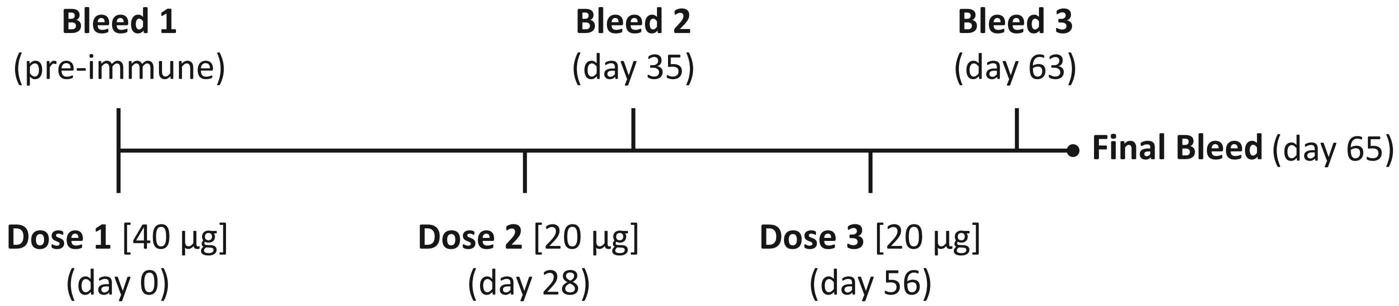

Injection scheme and blood sample time points of rat immunization with either DBL1x-2x or DBL3x-4ε, expressed in CHO cells or SHuffle bacteria, in combination with **(A)** physiological buffer (no adjuvant), Alhydrogel, GLA-SE, Freund's adjuvant or with **(B)** AbISCO®-100.

Table S1. Crude productivity (mg/L) and purity (%)

Proteins	CHO cells	*E. Coli* (SHuffle®)	*E. Coli* (HMS174)	*E. Coli* (BL21)	*Pichia pastoris*	*Lactococcus lactis*
DBL1x-6ε (3D7)	5 (1%)	ND	ND	ND	ND	ND
DBL1x-3x (3D7)	5 (1%)	26 (3%)	Low expressin	Low expression	No expression	ND
ID1-3x (3D7)	5 (1%)	30 (3%)	Low expression	Low expression	No expression	ND
DBL1x-2x (3D7)	**67 (13%)**	**133 (15%)**	65 (7%)	12 (3%)	2 (ND)	ND
DBL3x-4ε (FCR3)	**73 (18%)**	**96 (10%)**	Low expression	Low expression	3 (ND)	No expression

Table S2. Expression parameters

Growth Parameters				Induction Parameters			
**Test**	**Strain**	**Medium**	**T°**	**A600**	**T°**	**[IPTG]**	**Duration**
1	SHuffle	GY	30°C	2	20°C	0,1 mM	o/n
2	BL21	GY	30°C	2	20°C	0,1 mM	o/n
3	HMS174	GY	30°C	2	20°C	0,1 mM	o/n
4	BL21	GGLYP20	37°C	2	20°C	/	o/n
5	BL21	GY	37°C	2	30°C	0,01 mM	4-6 h
6	BL21	GY	30°C	2	30°C	0,01 mM	4-6 h

Table S3. Percent Identities of the DBL1x-6ε, DBL1x-2x and DBL3x-4ε obtained after ClustalW alignment of the 3D7, FCR3 and 7G8 sequences

	**DBL1-6 (Exon I)**	**DBL1-2**	**DBL3-4**
**3D7 vs FCR3**	81.7	82.2	87.0
**3D7 vs 7G8**	82.0	80.9	91.1
**FCR3 vs 7G8**	83.5	80.2	92.4
